# Short-chain dehydrogenases in *Haemonchus contortus*: changes during life cycle and in relation to drug-resistance

**DOI:** 10.1186/s13567-023-01148-y

**Published:** 2023-03-07

**Authors:** Karolína Štěrbová, Nikola Rychlá, Petra Matoušková, Lenka Skálová, Lucie Raisová Stuchlíková

**Affiliations:** Department of Biochemical Sciences, Faculty of Pharmacy, Charles University, Heyrovského, 1203 Hradec Králové, Czech Republic

**Keywords:** SDRs, *Haemonchus contortus*, expression profile, drug-susceptible strain, drug-resistant strain, phylogenetic analysis

## Abstract

Short-chain dehydrogenases/reductases (SDRs) regulate the activities of many hormones and other signaling molecules and participate in the deactivation of various carbonyl-bearing xenobiotics. Nevertheless, knowledge about these important enzymes in helminths remains limited. The aim of our study was to characterize the SDR superfamily in the parasitic nematode *Haemonchus contortus*. Genome localization of SDRs was explored, and phylogenetic analysis in comparison with SDRs from free-living nematode *Caenorhabditis elegans* and the domestic sheep (*Ovis aries*, a typical host of *H. contortus*) was constructed. The expression profile of selected SDRs during the life cycle along with differences between the drug-susceptible and drug-resistant strains, were also studied. Genome sequencing enabled the identification of 46 members of the SDR family in *H. contortus*. A number of genes have no orthologue in the sheep genome. In all developmental stages of *H. contortus*, SDR1, SDR3, SDR5, SDR6, SDR14, and SDR18 genes were the most expressed, although in individual stages, huge differences in expression levels were observed. A comparison of SDRs expression between the drug-susceptible and drug-resistant strains of *H. contortus* revealed several SDRs with changed expression in the resistant strain. Specifically, SDR1, SDR12, SDR13, SDR16 are SDR candidates related to drug-resistance, as the expression of these SDRs is consistently increased in most stages of the drug-resistant *H. contortus*. These findings revealing several SDR enzymes of *H. contortus* warrant further investigation.

**Supplementary Information:**

The online version contains supplementary material available at 10.1186/s13567-023-01148-y.

## Introduction

Short-chain dehydrogenases/reductases (SDRs) constitute one of the oldest and largest enzyme superfamilies, containing hundreds of thousands of members [1]. In phylogenetic comparisons, most members have only low pair-wise sequence identity, although they share common sequence motifs that define the cofactor binding site (TGxxxGxG) and the catalytic tetrad with highly conserved amino acids (Tyr, Lys, Ser, Asn). The three-dimensional SDR structures are homologous with a common α/β-folding pattern characterized by a central β-sheet typical of a Rossmann-fold with helices on either side. By contrast, the substrate recognition site, which is located at the C-terminus of the SDR protein, is highly variable between individual members, allowing for a broad substrate acceptance [2–4]. The majority of SDRs are oligomeric, with either homodimeric or homotetrameric quaternary structures. Monomeric SDRs such as carbonyl reductase (CBR) have a long segment of ~20 residues inserted just before the catalytic Tyr that forms an α-helix which packs against and stabilizes the helical interaction surface [5]. Many SDR family members are membrane-bound proteins with a predicted N-terminal transmembrane helix generally processing retinoid and steroid substrates [6].

SDR enzymes are NAD(P)(H)-dependent oxidoreductases present in all the genomes that have been investigated, from simple microorganisms to higher eukaryotes, a fact that emphasizes their versatility and fundamental importance for metabolic processes. In humans, over 70 SDR genes have been identified, and these enzymes are involved in the metabolism of a large variety of compounds, including steroid hormones, prostaglandins, retinoids, lipids, and xenobiotics. It is now clear that SDRs contribute to essential functions and interactions of all forms of life [4]. Nevertheless, no specific information about SDRs in parasitic nematodes has been made available.

Parasitic nematodes (also called roundworms) are dangerous pathogens of plants and animals, including humans. Parasitic nematodes infect over 25% of the human population and represent a major burden on livestock and crop production [7–9]. In animals, diseases caused by parasitic nematodes are accompanied by various types of clinical complications which cause permanent and long-term morbidity. Infections may occur in any tissue, but nematodes infecting the gastrointestinal tract and lungs are the most common and the most dangerous. Efficient and welfare-friendly livestock production demands constant and regular control of helminth infections. Pharmacotherapy of animals using various anthelmintic drugs represents the basic strategy for the treatment of helminth infections [10]. Despite the proliferation of drug-based treatment, only a limited number of anthelmintics are available on the market. Moreover, the effectiveness of these available drugs is limited, and the control of helminth infections is threatened due to increasing drug resistance in nematode populations [11]. It is therefore of utmost importance to develop new anthelmintic drugs, especially substances with more pronounced efficacy in nematodes resistant to classical anthelmintics [9, 12]. Considerable efforts have been devoted to the development of a new drug with a novel mechanism of action. To this end, the search for new drug targets and the identification of essential enzymes (enzymatic chokepoints), the blocking of which results in helminth fatality, may accelerate the process of developing novel drug candidates [13].

From this point of view, SDRs represent a fascinating set of enzymes due to their crucial role in the signaling processes [5, 14, 15]. Moreover, several SDR members participate in the deactivation of drugs and other xenobiotics in various organisms, including nematodes [16], and they may contribute to drug-resistance development. For this reason, our present study was designed to characterize the SDR family in the model parasitic nematode *Haemonchus contortus.* We have explored all the putative SDR genes in the *H. contortus* genome and described the phylogenetic analysis we have undertaken. We have quantitatively compared constitutive transcriptional levels of twenty-three SDRs in eggs, larvae, and adults. In all developmental stages, SDRs expression was compared among isolates with different levels of resistance: the susceptible (ISE) and the benzimidazole-resistant (IRE) isolates.

## Materials and methods

### SDR gene sequences and phylogenetic analysis

The SDR genes we used, were downloaded from the *H. contortus* genome (PRJEB506) from Wormbase Parasite [17]. The genome search was done using BioMart tool and the InerPro domain (IPR002347) query filter. Furthermore, due to the ongoing annotation process of *H. contortus* genome, we used obtained sequences and performed BLAST search. Resulting 46 sequences were included in the subsequent analysis. Protein sequences were compared and typical motifs defining SDR family were identified. The topology prediction tool DeepTMHMM [18, 19]) was used for the prediction of transmembrane helices (the outcome is listed in Table 1, details are available in the Additional file 1). Phylogenetic analysis of 46 SDR genes from *H. contortus* was performed along with 70 *C. elegans* genes with putative oxidoreductase function downloaded from WormBase [20] as orthologues of the *Hco*_SDR genes (list of *Cel*_SDRs accession numbers are available in the Additional file 2). The evolutionary analysis was conducted in MEGA 7 software [21]. First, the translated amino acid sequences were aligned using the MUSCLE program, and the model selection tool recommended a “LG + G + I” model as best fitting for the respective group of sequences for phylogenetic comparison (*Le Gascuel* model [22], including a discrete Gamma distribution to model evolutionary rate differences among sites (5 categories + G, parameter = 1.5771. The rate variation model allowed for some sites to be evolutionarily invariable [(+ I), 0.2475% sites, Additional file 3]. The evolutionary history was inferred using the Maximum Likelihood method based on this model [22]. The bootstrap consensus tree inferred from 500 replicates [23] was taken to represent the evolutionary history of the taxa analysed [23]. The percentage of replicate trees in which the associated taxa clustered together in the bootstrap test (500 replicates) have been shown next to the branches [23]. Initial tree(s) for the heuristic search were obtained by applying the Neighbor-Joining method to a matrix of pairwise distances estimated using a JTT model. The analysis involved 116 amino acid sequences, with all positions showing less than 95% site coverage eliminated, i.e. fewer than 5% alignment gaps, missing data, and ambiguous bases were allowed at any position. A total of 202 positions were represented in the final dataset.

**Table 1:** **Summary of** ***Haemonchus contortus* SDR sequences**

Gene model number	Assigned number	Chromosome location	Strand	DeepTMHMM prediction
HCON_00081410	sdr1	chr3	F	TM
HCON_00130000	sdr2	chr4	F	globular
HCON_00119730	sdr3	chr4	R	globular
HCON_00148540	sdr4	chr5	F	TM
HCON_00163110	sdr5	chr5	R	globular
HCON_00131840	sdr6	chr5	R	TM
HCON_00097820	sdr7	chr4	R	globular
HCON_00131890	sdr8	chr5	R	TM
HCON_00095920	sdr9	chr4	R	globular
HCON_00045600	sdr10	chr2	R	TM
HCON_00046500	sdr11	chr2	F	globular
HCON_00049110	sdr12	chr2	R	globular
HCON_00015620	sdr13	chr1	R	globular
HCON_00023910	sdr14	chr1	F	globular
HCON_00053800	sdr15	chr2	F	globular
HCON_00062110	sdr16	chr2	F	globular
HCON_00106500	sdr17	chr4	R	globular
HCON_00108480	sdr18	chr4	F	globular
HCON_00120550	sdr19	chr4	F	globular
HCON_00149440	sdr20	chr5	R	globular
HCON_00059510	sdr21	chr2	R	globular
HCON_00066650	sdr22	chr3	R	globular
HCON_00163970	sdr23	chrX	F	globular
HCON_00008190	–	chr1	F	globular
HCON_00009800	–	chr1	R	globular
HCON_00012630	–	chr1	R	TM
HCON_00015580	–	chr1	F	globular
HCON_00027690	–	chr1	F	globular
HCON_00027700	–	chr1	F	globular
HCON_00039800	–	chr2	R	globular
HCON_00053030	–	chr2	R	globular
HCON_00053160	–	chr2	R	globular
HCON_00062090	–	chr2	F	globular
HCON_00099350	–	chr4	F	TM
HCON_00102400	–	chr4	F	globular
HCON_00124340	–	chr4	F	globular
HCON_00124350	–	chr4	F	globular
HCON_00124360	–	chr4	F	globular
HCON_00124370	–	chr4	F	globular
HCON_00124380	–	chr4	F	globular
HCON_00130300	–	chr4	F	globular
HCON_00146780	–	chr5	F	globular
HCON_00152280	–	chr5	R	TM
HCON_00154500	–	chr5	F	globular
HCON_00161950	–	chr5	R	TM
HCON_00181460	–	chrX	R	globular

Similarly, the phylogenetic comparison of *Hco*_SDRs along with sheep SDRs (*Oar*_SDRs) was performed. All sheep protein sequences were retrieved from the NCBI Protein sequence database [24]. SDR members were identified by BLAST using 70 human SDR sequences [6]. All sequences above 60% similarity were used and no filter for isoforms was applied (NON-REDUNDANT PROTEIN SEQUENCES). Multiple sequence alignments were calculated using the MUSCLE. For phylogenetic analysis the Maximum Likelihood method was used [25] based on the Whelan And Goldman model [26]. The tree with the highest log likelihood (−42688.5410) is shown. The percentage of trees in which the associated taxa clustered together is shown next to the branches. Initial tree(s) for the heuristic search were obtained by applying the Neighbor-Joining method to a matrix of pairwise distances estimated using a JTT model. A discrete Gamma distribution was used to model evolutionary rate differences among sites (5 categories (+ *G*, parameter = 2.4299)). The analysis involved 207 amino acid sequences. All positions with less than 95% site coverage were eliminated. That is, fewer than 5% alignment gaps, missing data, and ambiguous bases were allowed at any position. There were a total of 155 positions in the final dataset. Accession numbers, the abbreviation used and full phylogenetic tree can be found in the Additional files 4, 5 and 6.

### The parasites and their collection

In this study, different life stages of two isolates of *H. contortus* were used: an inbred susceptible-Edinburgh strain (ISE, MHco3) and an inbred resistant-Edinburg strain (IRE, MHco5) [27]. Sheep (as a host of *H. contortus*) were bred and slaughtered in agreement with Czech slaughtering rules for farm animals and the Protection of Animals from Cruelty Act No. 246/1992, Czech Republic. The experimental project was evaluated and approved by the Ethics Committee of the Ministry of Education, Youth and Sports (MSMT-25908/2019).

Parasite-free lambs (3–4 months old) were orally infected with 6000 third-stage larvae (L3) of the ISE or IRE strain of *H. contortus*. Five weeks after infection the faeces were collected to obtain the eggs and larvae. The eggs were isolated from faeces using three sieves of varying mesh sizes [28] and purified with a sucrose flotation technique followed by washing in tap water. The first-stage larvae (L1) were cultivated from isolated eggs in tap water at 27 °C for 24 h. The larvae L3 were produced from eggs by incubating humidified faeces from infected lambs at 27 °C for one week. To obtain exsheathed third-stage larvae (xL3), the L3 were exposed to 0.15% (v/v) of sodium hypochlorite (NaClO) for 20 min at 37 °C and washed three times in tap water [29]. Seven weeks after infection, the lambs were stunned and immediately exsanguinated. The lambs’ abomasa were removed and adult nematodes were obtained using the agar method [30]. The freshly isolated adult nematodes were washed in phosphate-buffered saline (pH 7.4) and separated by gender under a microscope.

### RNA extraction and cDNA synthesis

To obtain enough total RNA, 100 000 eggs, 100 000 L1, 30 000 L3, 30 000 xL3, 15 adult males, and 10 adult females of *H. contortus* were used per one sample. Four biological replicates from all developmental stages were placed separately into plastic tubes with 1 mL of TriReagent® (Molecular Research Centre, OH, USA) and stored at –80 °C. The samples were homogenized using the FastPrep-24 5G Homogenizer (MP Biomedicals, France) for four 30 s intervals with a speed of 6.5 m/s. The total RNA was extracted using TriReagent according to the manufacturer’s protocol. The purity and concentrations of RNA were determined spectrophotometrically at a wavelength of 260 and 280 nm using the NanoDrop ND-1000 UV—Vis Spectrophotometer (Thermo Fisher Scientific, MA, USA) and analysed by the Agilent 2100 Bioanalyzer on RNA Nano chips (Agilent Technologies, CA, USA). To remove DNA contamination, 4 µg RNA of each sample were treated with DNase I (NEB, UK) and diluted to a concentration of 0.1 µg/µL. Complementary DNA was synthesized from 0.5 µg RNA in 20 µL reactions using random hexamer primers and Protoscript II Reverse Transcriptase (NEB, UK) following the manufacturer’s protocol. First-strand DNA was diluted 10× and stored at −20 °C until further analysis.

### Quantitative PCR (qPCR)

Four biological replicates and two technical replicates of each sample were analysed in a qPCR assay performed in the 384-Well PCR Thermal Cycler; QuantStudioTM 6 Flex Real-Time PCR System (Applied Biosystems, CA, USA) with SYBR Green I detection. The final master mix volume of 8 µL per well was prepared from 5 ng cDNA, qPCR Xceed SG 1 step 2 × Mix Lo-ROX (IAB, Czech Republic) as well as both forward and reverse primers (final concentration 100 nM). The PCR run protocol started with a denaturation step (95 °C for 2 min), followed by 40 cycles of two-step amplification (95 °C for 15 s, 60 °C for 20 s) with fluorescence measurement at the end of each cycle. A melting curve protocol with a heating rate of 0.5 °C every 30 s from 60 °C to 95 °C was used to investigate the specificity of the qPCR reaction. Gene-specific amplification was confirmed by a single peak for each sample. For the normalization of the qPCR assay, a combination of the two reference genes glyceraldehyde-3P-dehydrogenase (gpd) and RNA polymerase II large subunit (ama) was used as recommended by Lecová et al. [31]. Sequences were selected based on the available transcriptomes from the project PRJEB1360, Accession ERP00217R [37]. The TPMs (transcript per million) across all stages and three isolates with different level of drug-resistance, were compared (data available in Additional file 7). The selection criteria involved, an abundant expression in any of the stage analysed, with focus on adults and female gut expression, and differences in TPMs among the isolates. Due to the lack of proper SDR nomenclature, the transcripts analysed were named sdr1-23 (Table 1). The primer sequences for quantification SDR transcripts were designed in Primer3 software and synthesized by Generi Biotech, Czech Republic (Additional file 10). Before the PCR analysis, the specificity of the primers was confirmed by the presence of single peak in the melting curve analyses, and the efficiency of the primers was calculated from the slope of standard curves obtained from serial dilutions (1:5, 5 points) (Additional file 10).

### Statistical analysis

The reported data are expressed as the mean ± S.D. (four biological replicates of each sample). Relative expression was calculated using the ΔΔCt method [32]. The statistical significance of gene expression differences between the sensitive and resistant strains was evaluated using Multiple t-tests, where the ISE samples were set to one. All results were processed using GraphPad.

## Results

### Sequence analysis, genome localization and phylogenetic analysis of SDRs

**Figure 1:**
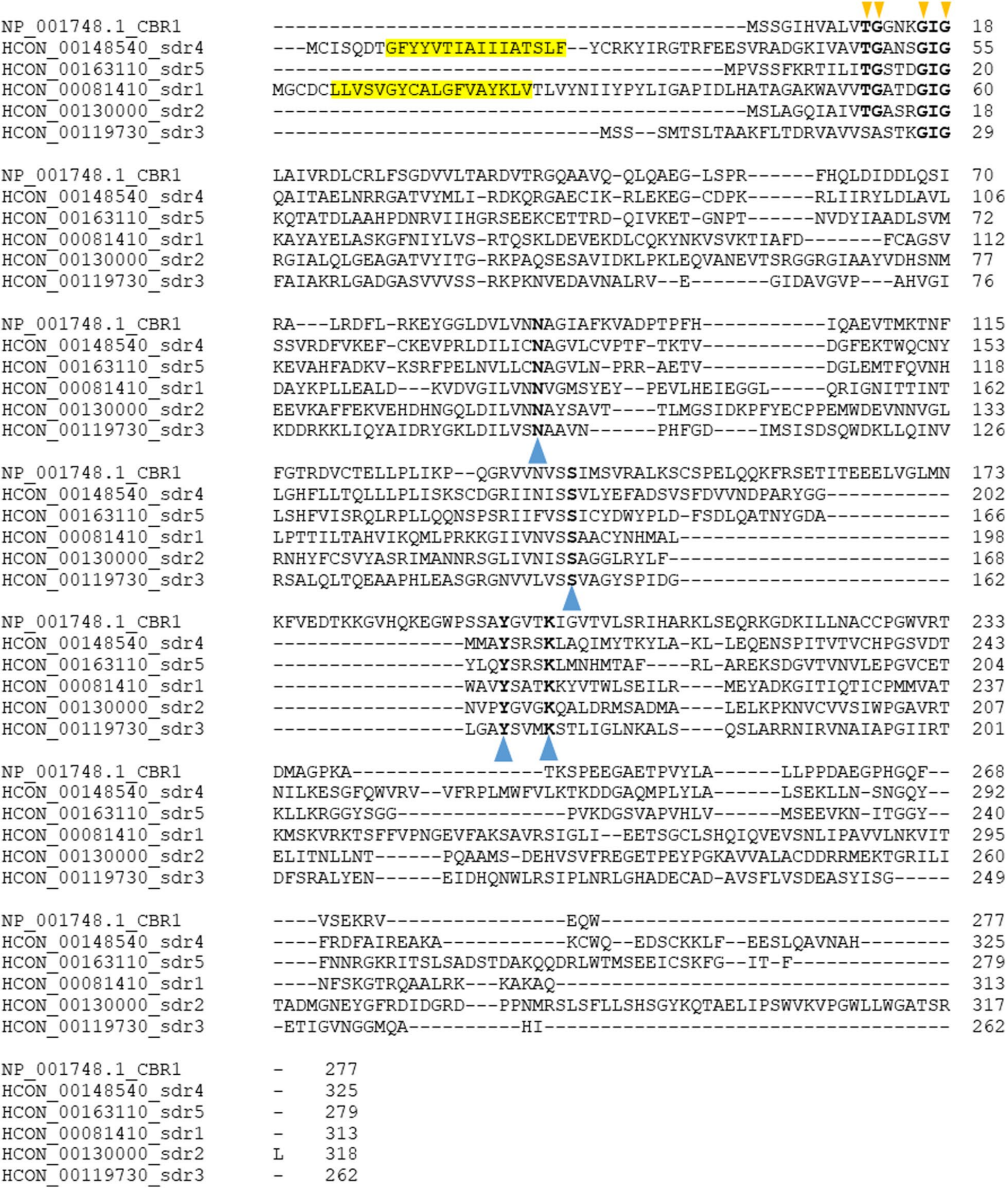
**The comparison of selected Hco_SDRs with human carbonyl reductase 1 (NP_001748.1-CBR1).** Multiple alignment was performed using Clustal Omega. Typical amino acids binding NAD(P)H are marked by orange triangles above the alignment, amino acids of the active site are marked by blue triangles bellow the alignment and the predicted transmembrane domain in N-terminus is located in yellow box.

Forty-six retrieved sequences from *H. contortus* genome were translated, aligned and searched for common sequence motifs that define SDRs (Figure 1, Additional file 8). The classical cofactor binding site motif (TGxxxGxG) was found in all *Hco*_SDRs but six; in HCON_00154500 and SDR22 (HCON_00066650) threonine is replaced by isoleucine and serin, respectively, in SDR3 (HCON_00119730) and HCON_00146780 the first glycine of the motif is replaced by alanine, in HCON_00015580 is the second glycine replaced by serine. In SDR7 (HCON_00097820) the motif corresponds with the “extended” SDR family (TGADGTIG) typical for epimerases and hydratases. The topology prediction tool DeepTMHMM predicted nine proteins having a C-terminal transmembrane domain (Table 1, Additional file 1).

**Figure 2:**
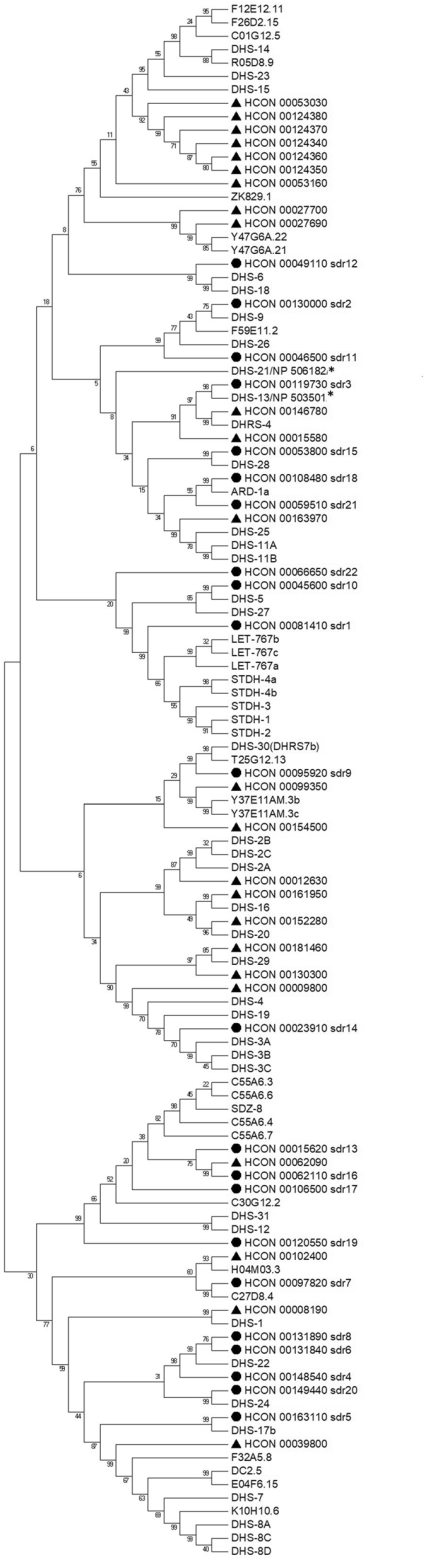
**Phylogenetic tree of** ***Haemonchus contortus*** **and** ***Caenorhabditis elegans*** **short-chain dehydrogenases (SDRs)**. A consensus phylogenetic tree was constructed using the Maximum Likelihood method based on the Le Gascuel 2008 model [19] in MEGA7. The bootstrap consensus tree was calculated (500 replicates), and reproduced partitions are denoted above branches [23]. *Hco*_SDRs analysed are marked by black dot, other *Hco*_SDRs are marked by black triangle, SDRs from *C. elegans* with reported function are marked by *.

**Figure 3:**
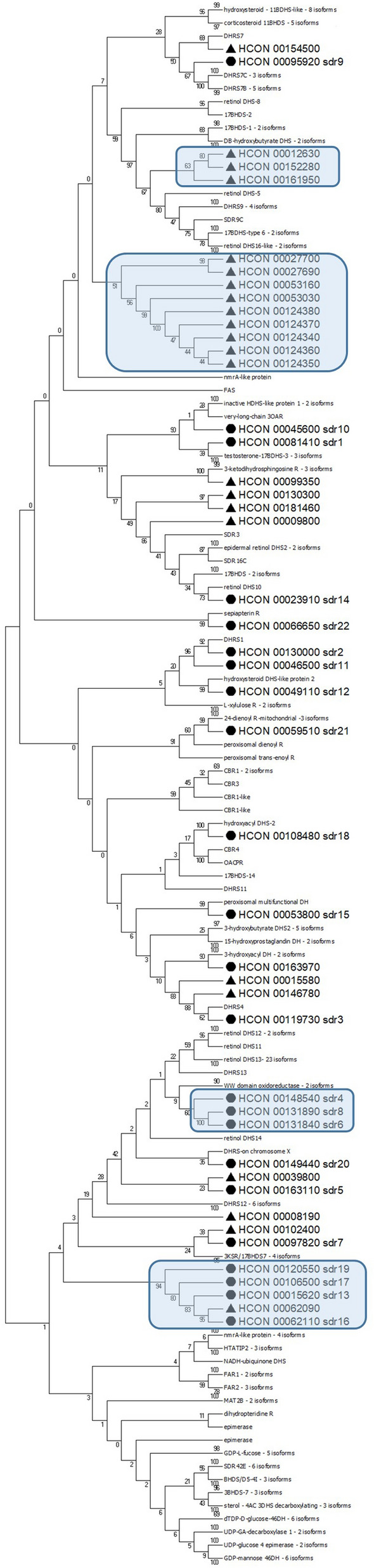
**Phylogenetic tree of** ***Haemonchus contortus*** **and* Ovis aries*** **short-chain dehydrogenases (SDRs)**. A consensus phylogenetic tree was constructed using the Maximum Likelihood method based on the Whelan And Goldman model [26] in MEGA7. Initial tree is display, the bootstrap consensus tree was calculated (100 replicates), partitions are denoted above branches. *Hco*_SDRs analysed are marked by black dot, other *Hco*_SDRs are marked by black triangle, expanded branches with several gene duplications potential as drug targets are in blue boxes. Sheep isoforms are collapsed, with mentioned number of isoforms (full details in Additional file 6).

Phylogenetic analysis was performed along with SDRs from *C. elegans* and sheep (*Ovis aries*). The *C. elegans* and *H. contortus* SDR gene families exist in monophyletic clades, suggesting an independent expansion of gene families within each species, having one-to-many orthologues in both directions; for example, ZK829.1 from *C. elegans* cluster with six *H. contortus* genes or HCON_0053160 cluster with seven genes from *C. elegans*. However, also a few one-to-one orthologues occur between the two nematode species suggesting ancient role of these genes (Figure 2). Interestingly, the phylogenetic analysis of *H. contortus* SDRs together with sheep proteins resulted in a similar outcome, suggesting more common SDRs ancestors before the organisms split. Four identified monophyletic clades of *H. contortus* sequences (highlighted in Figure 3) could be further explored as potential candidates for drug design.

**Figure 4:**
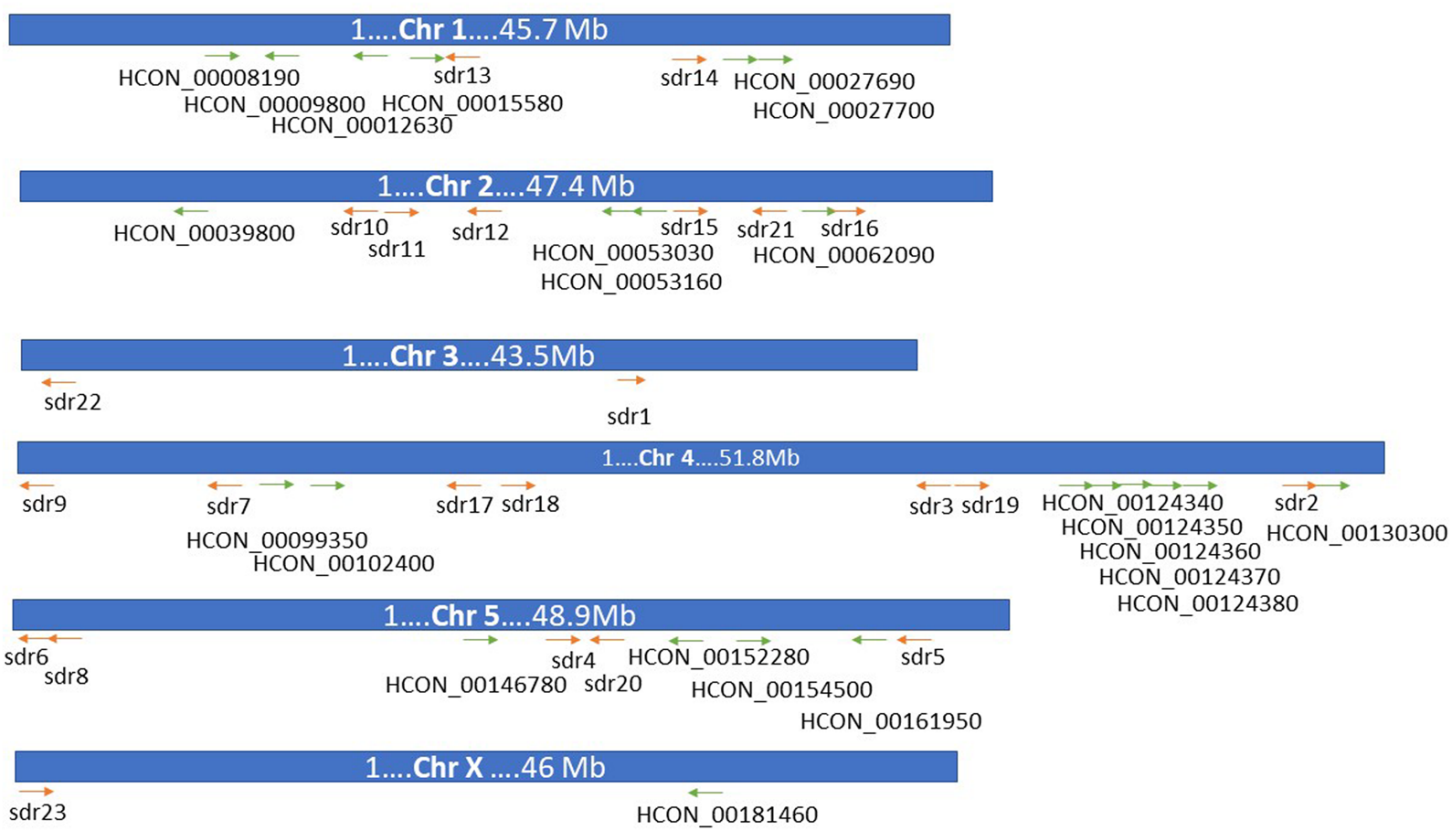
**Chromosome localization of each SDR gene.** The arrow direction indicates the location on forward ( →) or reverse ( ←) strand, arrows in red display the SDRs analysed in this study.

The distribution of SDR genes across *H. contortus* chromosomes is relatively even (Figure 4), with just chromosomes 3 and X containing only two SDR sequences. Most of the sequences (15) are located on chromosome 4, followed by chromosome 2 and 5 (10 and 9 sequences, respectively).

### SDRs expression in individual life stages

**Figure 5:**
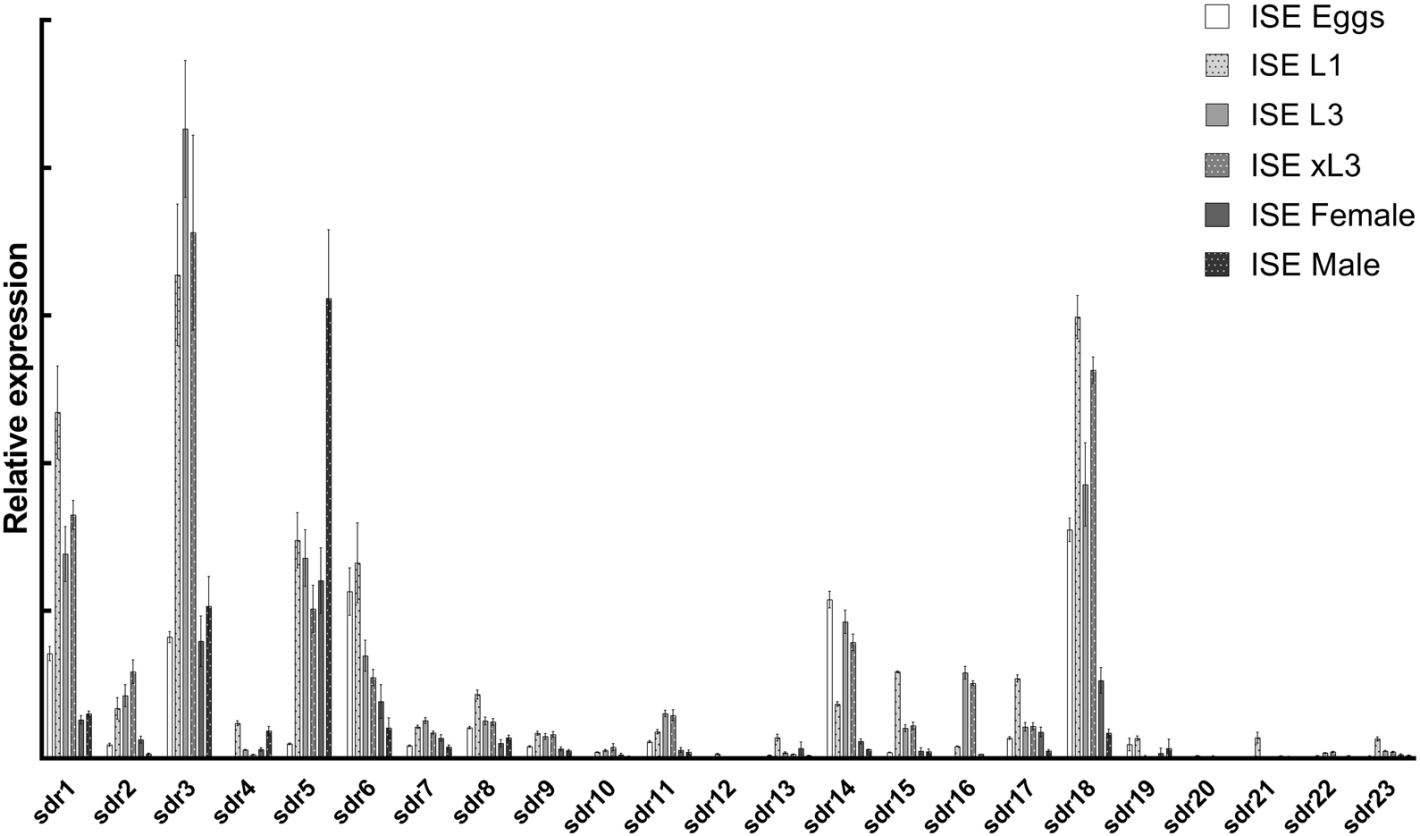
**Relative abundances of SDR genes in different life stages of sensitive ISE strain of** ***H. contortus***. For each SDR, the mean of relative expression is displayed as ΔCq, normalized to geometric mean of two reference genes (gdp, ama).

In *H. contortus* eggs, larvae (L1, L3 and xL3), and adults, the relative expression of twenty- three selected SDRs was quantified and compared, with the results presented in Figure 5. The expression differed significantly among individual SDRs as well as among life stages. In the eggs, SDR6, SDR14, and SDR18 represent the most abundant SDR transcripts, while SDR1, SDR3, and SDR18 dominate in the larvae. In the adults of both sexes, SDR3, SDR5, and SDR18 showed the highest expression. The expression of SDR5 was higher in the males than females, although the expression of SDR18 was higher in females, with a similar level of SDR3 detected in both sexes. Overall, most of SDRs exhibited the highest expression in free-living larvae. Only one, SDR5, was more highly expressed in the adult males than in any of the juvenile stages. In Additional file 9, a comparison of the transcriptome sequencing result and our qPCRs from the individual stage can be found.

### Comparison of SDRs expression in the ISE and IRE strain

**Figure 6:**
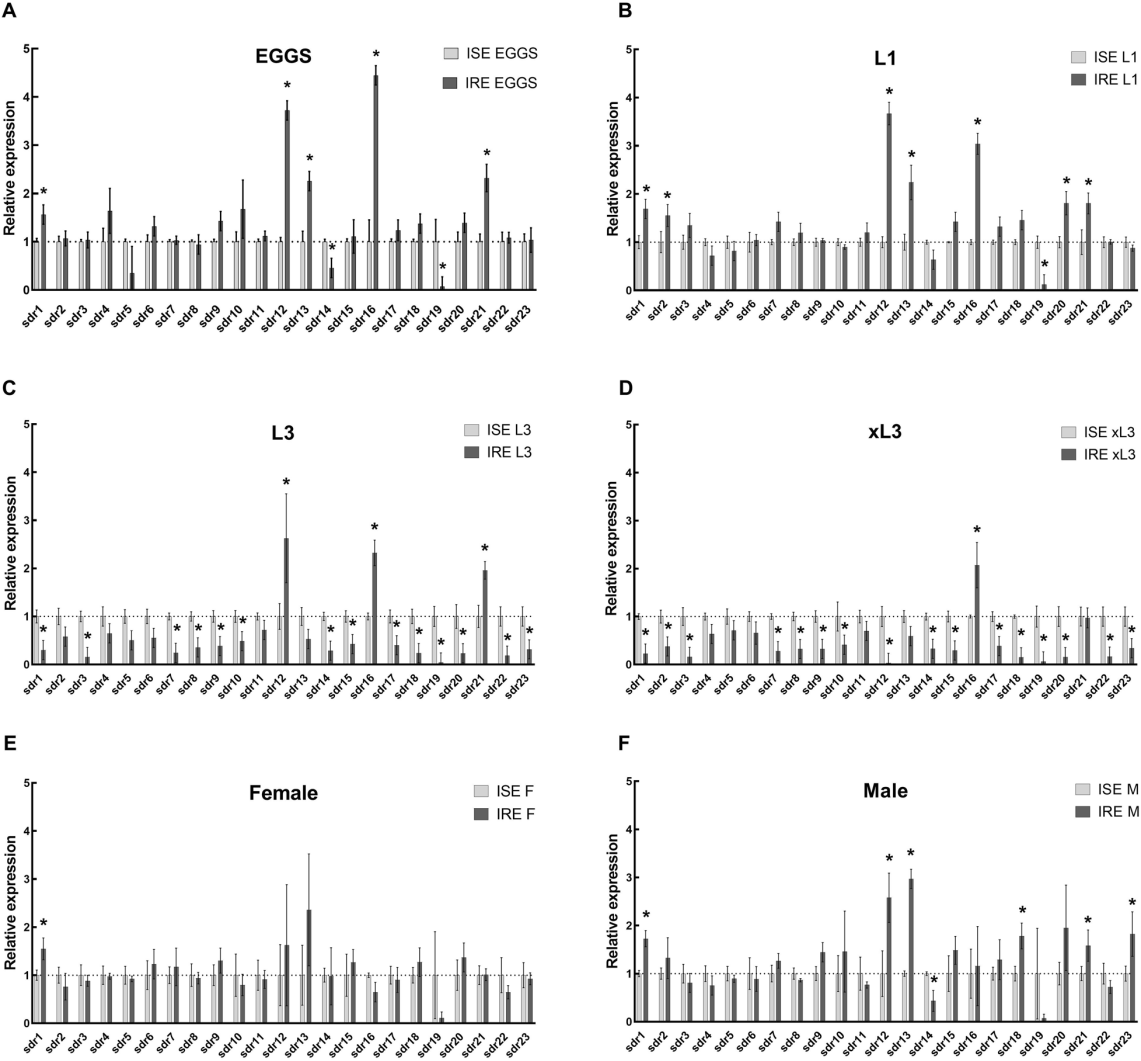
**The comparison of constitutive expression of SDR genes in** ***H. contortus*** **ISE and IRE strains analysed by qPCR**. Relative expression of selected *Hco*_SDRs mRNA in eggs (**A**), L1s (**B**), L3s (**C**), exL3s(**D**), adult females (**E**), adult males (**F**). * indicate significant difference between ISE and IRE at *P* > 0.05, *N* = 4.

The relative transcriptomic levels of individual SDRs in the *H. contortus* drug-susceptible strain ISE and the drug-resistant strain IRE were quantified and compared (see Figures 6A–F). In the eggs of the IRE strain, a significantly higher expression of SDR1, SDR12, SDR13, SDR16, and SDR21 was detected than was the case with the eggs of the ISE strain. On the other hand, two SDRs (SDR14 and SDR19) exhibited a higher expression in the ISE eggs than in the IRE eggs. In larvae L1 of the IRE strain, the same SDRs as in the eggs (i.e. SDR1, SDR12, SDR13, SDR16, and SDR21) were upregulated. In addition, the expression of SDR2 and SDR20 was also higher in L1 of the IRE strain than in L1 of the ISE strain. Only one transcript, SDR19, was detected at a lower level in L1 larvae of the IRE than the ISE strain. Surprisingly, in L3 and xL3 larvae of the IRE strain, most of the SDRs were downregulated in comparison to the ISE strain. Only three genes, SDR12, SDR16 and SDR21, were expressed at a higher level in L3 of the IRE than the ISE strain. In xL3, only SDR16 exhibited a higher expression in the IRE than in the ISE strain. In the adult males of the IRE strain, SDR1, SDR12, SDR13, SDR18, SDR21 and SDR23 were upregulated, while SDR14 and SDR19 were downregulated in comparison to the ISE strain. In the females, the expression of only SDR1 was significantly higher in the IRE than in the ISE strain, with other differences in SDRs expression levels between IRE and ISE strains found to be insignificant due to large standard deviations.

## Discussion

The successful battle against helminths, including *H. contortus,* requires deeper knowledge of their physiology and biochemistry [33]. The molecular analyses of key developmental, metabolic and structural process of *H. contortus* is necessary to identify new targets of potential anthelmintics [34]. Although SDRs are essential enzymes in almost all organisms, no information about this superfamily in the parasitic nematode *H.* *contortus* has been made available.

To begin to fill this knowledge gap, we analysed the SDR family in *H. contortus*. The results revealed 46 members of the SDR superfamily. The sequence comparison revealed the typical cofactor binding site motif (TGxxxGxG) in most of the *Hco*_SDRs. The exceptions were SDR3 (HCON_00119730) and HCON_00146780, in which the first glycine of the motif is replaced by alanine, as is the case in a homologue DHS-13 from *C. elegans* and DHRS4 in sheep involved in the metabolism of aromatic carbonyl compounds [3]. Furthermore, in SDR7 (HCON_00097820) the motif slightly differs (TGADGTIG), corresponding to the sheep/human homologue HTATIP2, an atypical member of the SDRs superfamily. The localization of SDRs in the genome of *H. contortus* was undertaken, and a phylogenetic tree was created with a focus on the comparison of SDRs in *H. contortus* with the free-living nematode *C. elegans* and sheep as a common host of *H. contortus.* The phylogenetic tree shows several genes to have a one-to-one homologue in *H. contortus* and *C. elegans*, suggesting ancestral role in the organisms. On the other hand, several genes show one-to-many homologues in both directions, suggesting specific roles of multiplied SDRs in the respective organism. The largest cluster of *H. contortus* SDR genes are located on chromosome 4, previously shown to have a high recombination rate [35] and also to contain most of the UDP-glycosyltransferases (20 genes), another important enzyme superfamily with a signaling and detoxification function [36].

Interestingly, five genes located in one cluster on chromosome 4 (HCON_00124340-80), clearly originating from gene duplication, cluster best with another gene (HCON__00053030) located on chromosome 2. Only one close *C. elegans* orthologue, ZK829.1, was found, a finding which suggests the involvement of respective SDR enzymes in the parasitic lifestyle. These five genes along with four other genes form the largest monophyletic clade in the phylogenetic comparison with sheep SDRs having no direct sheep orthologue. On the other hand, four genes (HCON_00062090, _00062110, _00015620, _00106500) cluster together despite their presence on three different chromosomes. These four genes, among others cluster with the SDZ-8 *C. elegans* gene which is under control of SKN1 transcription factor [37], the oxidative stress-inducible transcription factor involved in xenobiotic detoxification and ageing [38]. Therefore, these four genes might be considered as essential players in the detoxification of xenobiotics, a supposition which is well supported by higher expression of two of these SDRs, SDR13 and SDR16 (HCON_00062110, _00015620, respectively), in several life stages of the resistant *H. contortus* strain.

To find out more about SDRs in *H. contortus*, 23 SDR sequences were selected for thorough expression analysis based on TPMs (transcript per million) from transcriptomes available within the project PRJEB1360, Accession ERP00217R [34]. Those sequences which showed an abundant expression in any of the *H. contortus* stages (eggs, L1, L3, adults) or had higher expression in the gut were selected. We mostly aimed to focus on the SDRs that are expressed in adults (as a drug target stage) and in the gut, as there is higher chance of these SDRs being targeted by novel potential drugs. Due to the lack of proper SDR nomenclature, the transcripts analysed were named SDR1-23 (in the Table 1, the gene model numbers and primers are provided in full details). In Additional file 9, a comparison of the publicly available transcriptome sequencing results and our qPCRs from individual stages can be found.

Comparing individual SDRs among the life stages, the relative expression differed significantly; while the transcription of some SDRs reached a high level in all living stages, the expression of other SDRs was barely detectable. The levels of most of the SDRs during development well correlate with transcriptomic data reported previously [39]. Comparing changes in the expression level of individual SDRs during the life cycle, the expression of only one enzyme (SDR5) was shown to increase continuously from eggs to larvae and adults, and one enzyme (SDR6) decreases from eggs to larvae and adults; although the expression of the most enzymes is low in the eggs, reaching the highest level in larvae, it decreases in adults. Overall, most SDRs exhibited the highest expression in free-living larvae, which indicates an important function of these enzymes during larvae life in an external environment. Comparing L1, L3 and xL3, some changes in expression of individual SDRs were observed, therefore the importance and function of individual SDRs probably also changes during larvae development. The expression of only SDR5 was higher in adult males (the stage living within the host and feeding on blood) than in any of the juvenile stages.

Developmental changes in SDRs expression in *H. contortus* likely reflect the adaptation to different life conditions. While aerobic metabolism of larvae depends on an efficient oxidative phosphorylation, the anaerobic metabolism of adults requires glycolysis, resulting in the production of volatile fatty acids such as acetic acid and propionic acid. In addition, adults have reduced pathways for amino acid synthesis, purine and pyrimidine salvage pathways as well as lipid metabolism [34]. Moreover, contrary to parasitic adults, free-living stages need efficient defenses against potentially harmful xenobiotics present in the environment. On the other hand, parasitic adults must deal with the defensive immune system of the host. It can be assumed that SDRs participate in these processes, although a deeper understanding of their function in individual developmental stages will require many further studies.

With the aim of evaluating the relationship between SDRs and drug-resistance, the transcriptomic levels of individual SDRs in the *H. contortus* drug-susceptible strain ISE and drug-resistant strain IRE were compared. In the eggs, L1 larvae and adult males of the IRE strain, expression of several SDRs (primarily SDR1, SDR12, SDR13, and SDR16) was increased, with only one gene (SDR19) decreased in comparison to the ISE strain. The initial selection of SDR transcripts for our experimental analysis involved also the differences in TPMs between sensitive ISE strain and two ivermectin-resistant strains CAVR and WR from the analysis of adult females (Additional file 7), where many SDRs were expressed differently among the strains. In our experiments only one transcript (SDR1) showed higher expression in benzimidazoles-resistant females (IRE), which is not surprising, since ISE and IRE are genetically closer than ISE and CAVR or WR that are genetically divergent strains of *H. contortus* with very high level of inter-strain transcriptional diversity [40]. Surprisingly, in L3 larvae of the IRE strain, most of the SDRs were decreased, with only three genes, SDR12, SDR16 and SDR21, increased in comparison to the ISE strain. Following these results, SDR1, SDR12, SDR13, SDR16, SDR14, SDR19 and SDR21 may be considered as candidate SDRs in terms of drug-resistance, as the expressions of these SDRs in almost all stages of the *H. contortus* IRE strain were consistently increased or decreased, respectively.

Although, only constitutive expression of SDRs was analysed and compared we infer their involvement in drug-resistance, since as we reported previously the reduction of anthelmintic flubendazole is higher in the IRE strain [41]. Indeed, we cannot exclude the possibility that this difference is based on a more potent induction of some SDR transcripts by the anthelmintic rather than a difference in the constitutive expression. However, only by functional analysis can the involvement of candidate genes in the resistance be confirmed.

In conclusion, our results, together with the above-mentioned information from literature demonstrate the need for a deeper study of SDRs in helminths. In the *H. contortus* genome 46 SDR genes were identified, some of which highly expressed in all developmental stages and some expressed in higher amount in the resistant strain. Therefore, several members of the *H. contortus* SDR family warrant further investigation as potential drug targets as well as in terms of their potential role in drug resistance.

## Supplementary Information

**Additional file 1: DeepTMHMM—Predictions.**

**Additional file 2: List of C. elegans SDR genes available in WormBase.**

**Additional file 3: Additional file 3 Model selection for phylogenetic analysis of Hco_SDRs and Cel_SDRs for Maximum Likelihood fits of 56 different amino acid substitution models.**

**Additional file 4: List of sheep SDR proteins available in NCBI.**

**Additional file 5: Maximum Likelihood fits of 56 different amino acid substitution models.**

**Additional file 6: Phylogenetic tree of *Haemonchus contortus*** **and** ***Ovis aries*** **short-chain dehydrogenases (SDRs) in full details**. A consensus phylogenetic tree was constructed using the Maximum Likelihood method based on the Whelan And Goldman model [27] in MEGA7. Initial tree is display, the bootstrap consensus tree was calculated (100 replicates), partitions are denoted above branches. *Hco*_SDRs analysed are marked by black dot, other *Hco*_SDRs are marked by black triangle.

**Additional file 7: Transcript per milion (TPMs) from RNA-seq for *H. contortus*** **SDRs**.

**Additional file 8: The comparison of all Hco_SDRs**. Multiple alignment was performed using MultAlin [44]. Red residues represent high (>90%) consensus level and blue residues represent low (>50%) consensus level.

**Additional file 9: The comparison of expression levels of SDRs based on the relative quantification by qPCR (left axis, circles and dotted line) and relative quantification based on TPM values (transcript per million - right axes, squares and full line) all TPM data for SDRs available in Additional file 2) by differential RNA sequencing **[39]**).**

**Additional file 10: Primers for qPCR.**

## References

[1] Short-chain Dehydrogenases/Reductases Databases. http://www.sdr-enzymes.org/

[2] Beck KR, Kaserer T, Schuster D, Odermatt A (2017). Virtual screening applications in short-chain dehydrogenase/reductase research. J Steroid Biochem Mol Biol.

[3] Kisiela M, Faust A, Ebert B, Maser E, Scheidig AJ (2018). Crystal structure and catalytic characterization of the dehydrogenase/reductase SDR family member 4 (DHRS4) from *Caenorhabditis elegans*. Febs J.

[4] Persson B, Kallberg Y, Bray JE, Bruford E, Dellaporta SL, Favia AD, Duarte RG, Jornvall H, Kavanagh KL, Kedishvili N, Kisiela M, Maserk E, Mindnich R, Orchard S, Penning TM, Thornton JM, Adamski J, Oppermann U (2009). The SDR (short-chain dehydrogenase/reductase and related enzymes) nomenclature initiative. Chem Biol Interact.

[5] Kavanagh K, Jornvall H, Persson B, Oppermann U (2008). The SDR superfamily: functional and structural diversity within a family of metabolic and regulatory enzymes. Cell Mol Life Sci.

[6] Bray JE, Marsden BD, Oppermann U (2009). The human short-chain dehydrogenase/reductase (SDR) superfamily: a bioinformatics summary. Chem Biol Interact.

[7] Patil KD, Bagade SB, Sharma SR, Hatware KV (2019). Potential of herbal constituents as new natural leads against helminthiasis: a neglected tropical disease. Asian Pac J Trop Med.

[8] Idris OA, Wintola OA, Afolayan AJ (2019). Helminthiases; prevalence, transmission, host-parasite interactions, resistance to common synthetic drugs and treatment. Heliyon.

[9] Zajickova M, Nguyen LT, Skalova L, Stuchlikova LR, Matouskova P (2020). Anthelmintics in the future: current trends in the discovery and development of new drugs against gastrointestinal nematodes. Drug Discov Today.

[10] Stear MJ, Doligalska M, Donskow-Schmelter K (2007). Alternatives to anthelmintics for the control of nematodes in livestock. Parasitology.

[11] Kotze AC, Prichard RK (2016). Anthelmintic resistance in *Haemonchus contortus*: history, mechanisms and diagnosis. Adv Parasitol.

[12] Lanusse C, Canton C, Virkel G, Alvarez L, Costa L, Lifschitz A (2018). Strategies to optimize the efficacy of anthelmintic drugs in ruminants. Trends Parasitol.

[13] Preston S, Jabbar A, Gasser RB (2016). A perspective on genomic-guided anthelmintic discovery and repurposing using *Haemonchus contortus*. Infect Genet Evol.

[14] Ferrandi EE, Bertuletti S, Monti D, Riva S (2020). Hydroxysteroid dehydrogenases: an ongoing story. Eur J Organ Chem.

[15] Hoffmann F, Maser E (2007). Carbonyl reductases and pluripotent hydroxysteroid dehydrogenases of the shortchain dehydrogenase/reductase superfamily. Drug Metab Rev.

[16] Matouskova P, Vokral I, Lamka J, Skalova L (2016). The role of xenobiotic-metabolizing enzymes in anthelmintic deactivation and resistance in helminths. Trends Parasitol.

[17] WormBase Parasite databases. https://parasite.wormbase.org

[18] Technical University of Denmark. https://dtu.biolib.com/DeepTMHMM/

[19] Hallgren J, Tsirigos KD, Pedersen MD, Almagro Armenteros JJ, Marcatili P, Nielsen H, Krogh A (2022). Winther O (2022) DeepTMHMM predicts alpha and beta transmembrane proteins using deep neural networks. bioRxiv.

[20] WormBase databases. https://wormbase.org

[21] Tamura K, Stecher G, Kumar S (2021). MEGA11 molecular evolutionary genetics analysis version 11. Mol Biol Evol.

[22] Le SQ, Gascuel O (2008). An improved general amino acid replacement matrix. Mol Biol Evol.

[23] Felsenstein J (1985). Confidence limits on phylogenies: an approach using the bootstrap. Evolution.

[24] National Library of Medicine. https://www.ncbi.nlm.nih.gov/10.1080/15360280801989377 28792816

[25] Kumar S, Stecher G, Tamura K (2016). MEGA7: molecular evolutionary genetics analysis version 7.0 for bigger datasets. Mol Biol Evol.

[26] Whelan S, Goldman N (2001). A general empirical model of protein evolution derived from multiple protein families using a maximum-likelihood approach. Mol Biol Evol.

[27] Roos MH, Otsen M, Hoekstra R, Veenstra JG, Lenstra JA (2004). Genetic analysis of inbreeding of two strains of the parasitic nematode *Haemonchus contortus*. Int J Parasitol.

[28] Varady M, Cudekova P, Corba J (2007). In vitro detection of benzimidazole resistance in *Haemonchus contortus*: Egg hatch test versus larval development test. Vet Parasitol.

[29] Nguyen LT, Kurz T, Preston S, Brueckmann H, Lungerich B, Herath HMPD, Koehler AV, Wang T, Skálová L, Jabbar A, Gasser RB (2019). Phenotypic screening of the ‘Kurz-box’ of chemicals identifies two compounds (BLK127 and HBK4) with anthelmintic activity in vitro against parasitic larval stages of *Haemonchus contortus*. Parasit Vectors.

[30] Vanwyk JA, Gerber HM, Groeneveld HT (1980). A technique for the recovery of nematodes from ruminants by migration from gastrointestinal ingesta gelled in agar-large scale application. Onderstepoort J Vet Res.

[31] Lecová L, Růžičková M, Laing R, Vogel H, Szotáková B, Prchal L, Lamka J, Vokřál I, Skálová L, Matoušková P (2015). Reliable reference gene selection for quantitative real time PCR in *Haemonchus contortus*. Mol Biochem Parasitol.

[32] Livak KJ, Schmittgen TD (2001). Analysis of relative gene expression data using real-time quantitative PCR and the 2(T)(-Delta Delta C) method. Methods.

[33] Morgan ER, Aziz NA, Blanchard A, Charlier J, Charvet C, Claerebout E, Geldhof P, Greer AW, Hertzberg H, Hodgkinson J, Hoglund J, Hoste H, Kaplan RM, Martinez-Valladares M, Mitchell S, Ploeger HW, Rinaldi L, von Samson-Himmelstjerna G, Sotiraki S, Schnyder M, Skuce P, Bartley D, Kenyon F, Thamsborg SM, Vineer HR, de Waal T, Williams AR, van Wyk JA, Vercruysse J (2019). 100 questions in livestock helminthology research. Trends Parasitol.

[34] Harder A, Samson-Himmelstjerna G (2016). The Biochemistry of *Haemonchus contortus* and Other Parasitic Nematodes. Gasser R.

[35] Doyle SR, Laing R, Bartley DJ, Britton C, Chaudhry U, Gilleard JS, Holroyd N, Mable BK, Maitland K, Morrison AA, Tait A, Tracey A, Berriman M, Devaney E, Cotton JA, Sargison ND (2018). A genome resequencing-based genetic map reveals the recombination landscape of an outbred parasitic nematode in the presence of polyploidy and polyandry. Genome Biol Evol.

[36] Matoušková P, Lecová L, Laing R, Dimunová D, Vogel H, Raisová Stuchlíková L, Nguyen LT, Kellerová P, Vokřál I, Lamka J, Szotáková B, Várady M, Skálová L (2018). UDP-glycosyltransferase family in *Haemonchus contortus*: phylogenetic analysis, constitutive expression, sex-differences and resistance-related differences. Int J Parasitol Drugs Drug Resist.

[37] Blackwell TK, Steinbaugh MJ, Hourihan JM, Ewald CY, Isik M (2015). SKN-1/Nrf, stress responses, and aging in *Caenorhabditis elegans*. Free Radic Biol Med.

[38] Park SK, Tedesco PM, Johnson TE (2009). Oxidative stress and longevity in *Caenorhabditis elegans* as mediated by SKN-1. Aging Cell.

[39] Laing R, Kikuchi T, Martinelli A, Tsai IJ, Beech RN, Redman E, Holroyd N, Bartley DJ, Beasley H, Britton C, Curran D, Devaney E, Gilabert A, Hunt M, Jackson F, Johnston SL, Kryukov I, Li K, Morrison AA, Reid AJ, Sargison N, Saunders GI, Wasmuth JD, Wolstenholme A, Berriman M, Gilleard JS, Cotton JA (2013). The genome and transcriptome of *Haemonchus contortus*, a key model parasite for drug and vaccine discovery. Genome Biol.

[40] Rezansoff AM, Laing R, Martinelli A, Stasiuk S, Redman E, Bartley D, Holroyd N, Devaney E, Sargison ND, Doyle S, Cotton JA, Gilleard JS (2019). The confounding effects of high genetic diversity on the determination and interpretation of differential gene expression analysis in the parasitic nematode *Haemonchus contortus*. Int J Parasitol.

[41] Stuchlíková LR, Matoušková P, Vokřál I, Lamka J, Szotáková B, Sečkařová A, Dimunová D, Nguyen LT, Várady M, Skálová L (2018). Metabolism of albendazole, ricobendazole and flubendazole in *Haemonchus contortus* adults: sex differences, resistance-related differences and the identification of new metabolites. Int J Parasitol Drugs Drug Resist.

[42] Multiple sequence alignment by Florence Corpet. http://multalin.toulouse.inra.fr/

[43] Nei M, Kumar S (2000). Molecular evolution and phylogenetics.

