# Supplementary material for: Short-chain dehydrogenases in Haemonchus contortus: changes during life cycle and in relation to drug-resistance
Source: Vet Res. 2023 Mar 7;54:19. doi: 10.1186/s13567-023-01148-y (PMC9993613; doi:10.1186/s13567-023-01148-y)
Supplement: Supplementary file 6 — Additional file 6: Phylogenetic tree of Haemonchus contortus and Ovis aries short-chain dehydrogenases (SDRs) in full details. A consensus phylogenetic tree was constructed using the Maximum Likelihood method based on the Whelan And Goldman model [27] in MEGA7. Initial tree is display, the bootstrap consensus tree was calculated (100 replicates), partitions are denoted above branches. Hco_SDRs analysed are marked by black dot, other Hco_SDRs are marked by black triangle. [file 13567_2023_1148_MOESM6_ESM.docx]

**Additional file 6**

Phylogenetic tree of *Haemonchus contortus* and *Ovis aries* short chain dehydrogenases (SDRs) in full. A consensus phylogenetic tree was constructed using the Maximum Likelihood method based on the Whelan And Goldman model [26] in MEGA7. Initial tree is display, the bootstrap consensus tree was calculated (100 replicates), partitions are denoted above branches. *Hco*_SDRs analysed are maked by black dot, other *Hco*_SDRs are marked by black triangle.


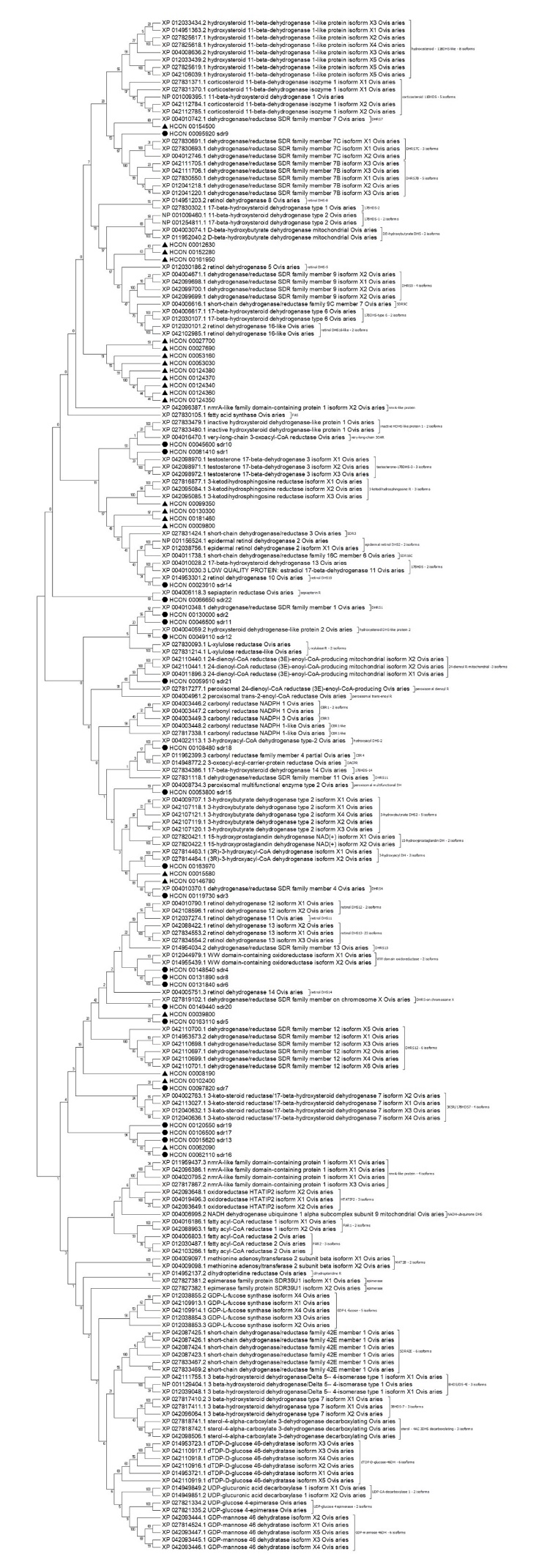


All sheep protein sequences were retrieved from the NCBI Protein sequence database [24]. SDR members were identified by BLAST using all the human seuences [23]. All sequences above 60% similarity were used and no filter for isoforms was applied (NON-REDUNDANT PROTEIN SEQUENCES). Multiple sequence alignments were calculated using the MUSCLE. For phylogenetic analysis the Maximum Likelihood method was used [27] based on the Whelan And Goldman model [26]. The tree with the highest log likelihood (-42688.5410) is shown. The percentage of trees in which the associated taxa clustered together is shown next to the branches. Initial tree(s) for the heuristic search were obtained by applying the Neighbor-Joining method to a matrix of pairwise distances estimated using a JTT model. A discrete Gamma distribution was used to model evolutionary rate differences among sites (5 categories (+G, parameter = 2.4299)). The analysis involved 207 amino acid sequences. All positions with less than 95% site coverage were eliminated. That is, fewer than 5% alignment gaps, missing data, and ambiguous bases were allowed at any position. There were a total of 155 positions in the final dataset. Evolutionary analyses were conducted in MEGA7 [23].
