# Supplementary material for: Short-chain dehydrogenases in Haemonchus contortus: changes during life cycle and in relation to drug-resistance
Source: Vet Res. 2023 Mar 7;54:19. doi: 10.1186/s13567-023-01148-y (PMC9993613; doi:10.1186/s13567-023-01148-y)

**Additional file 8**

**Comparison of all *Hco*_SDRs.** Multiple alignment was performed using MultAlin [42]. Red residues represent high (>90%) consensus level and blue residues represent low (>50%) consensus level.


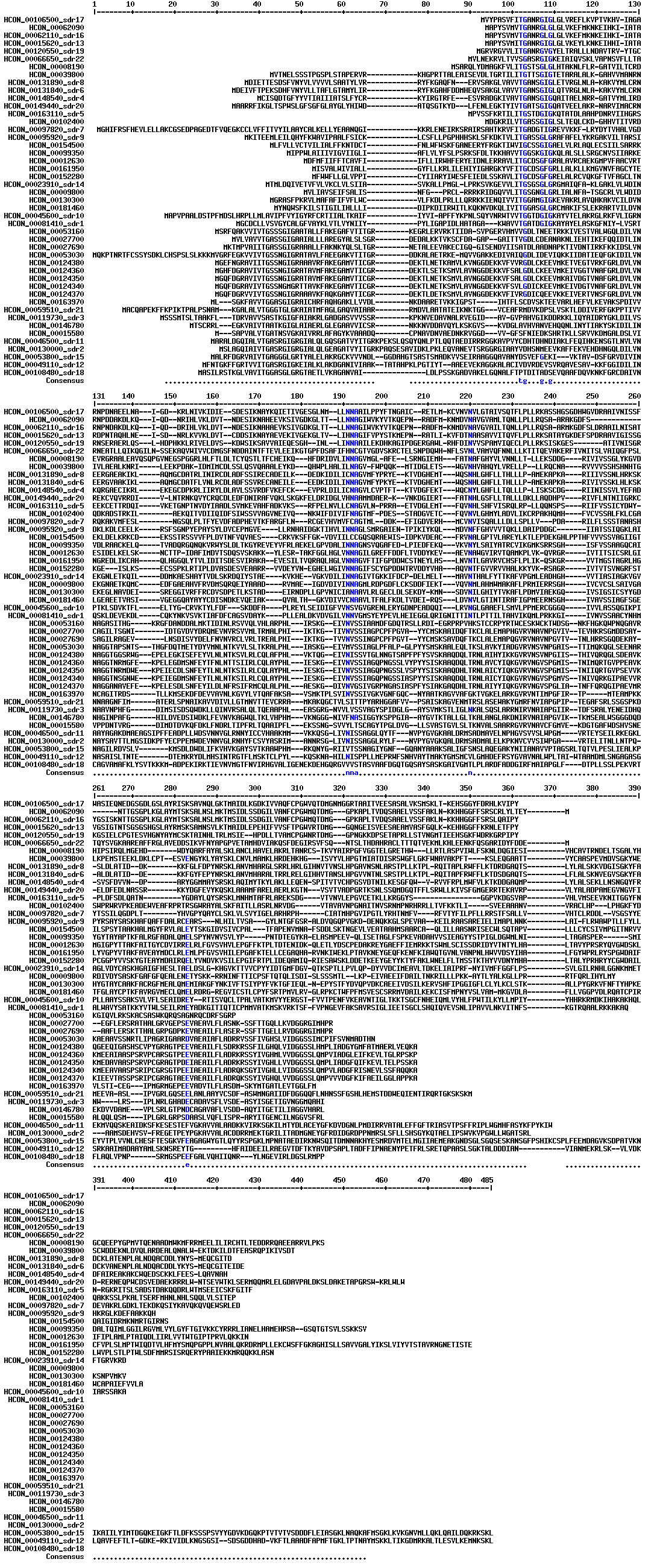

Supplement: Supplementary file 8 — Additional file 8: The comparison of all Hco_SDRs. Multiple alignment was performed using MultAlin [44]. Red residues represent high (>90%) consensus level and blue residues represent low (>50%) consensus level. [file 13567_2023_1148_MOESM8_ESM.docx]
