# Supplementary material for: Short-chain dehydrogenases in Haemonchus contortus: changes during life cycle and in relation to drug-resistance
Source: Vet Res. 2023 Mar 7;54:19. doi: 10.1186/s13567-023-01148-y (PMC9993613; doi:10.1186/s13567-023-01148-y)
Supplement: Supplementary file 9 — Additional file 9: The comparison of expression levels of SDRs based on the relative quantification by qPCR (left axis, circles and dotted line) and relative quantification based on TPM values (transcript per million - right axes, squares and full line) all TPM data for SDRs available in Additional file 2) by differential RNA sequencing [39]). [file 13567_2023_1148_MOESM9_ESM.docx]

**Additional file 9**

The comparison of expression levels of Hco_SDRs based on the relative quantification by qPCR (left axis, circles and dotted line) and relative quantification based on TPM values (transcript per million - right axes, squares and full line, all TPM data for SDRs available in supplementary table ST7) from differential RNA sequencing [39]).

**
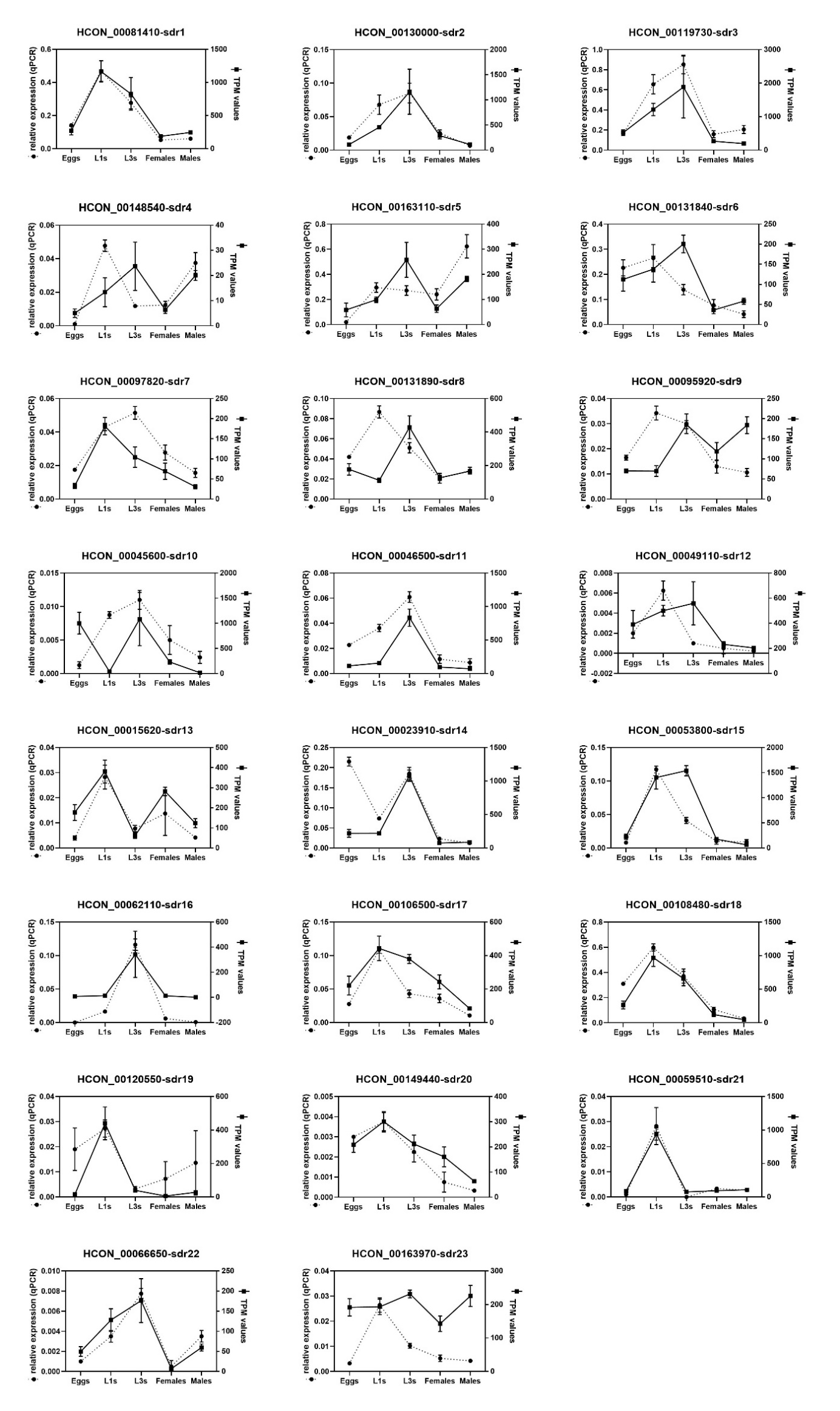
**

**
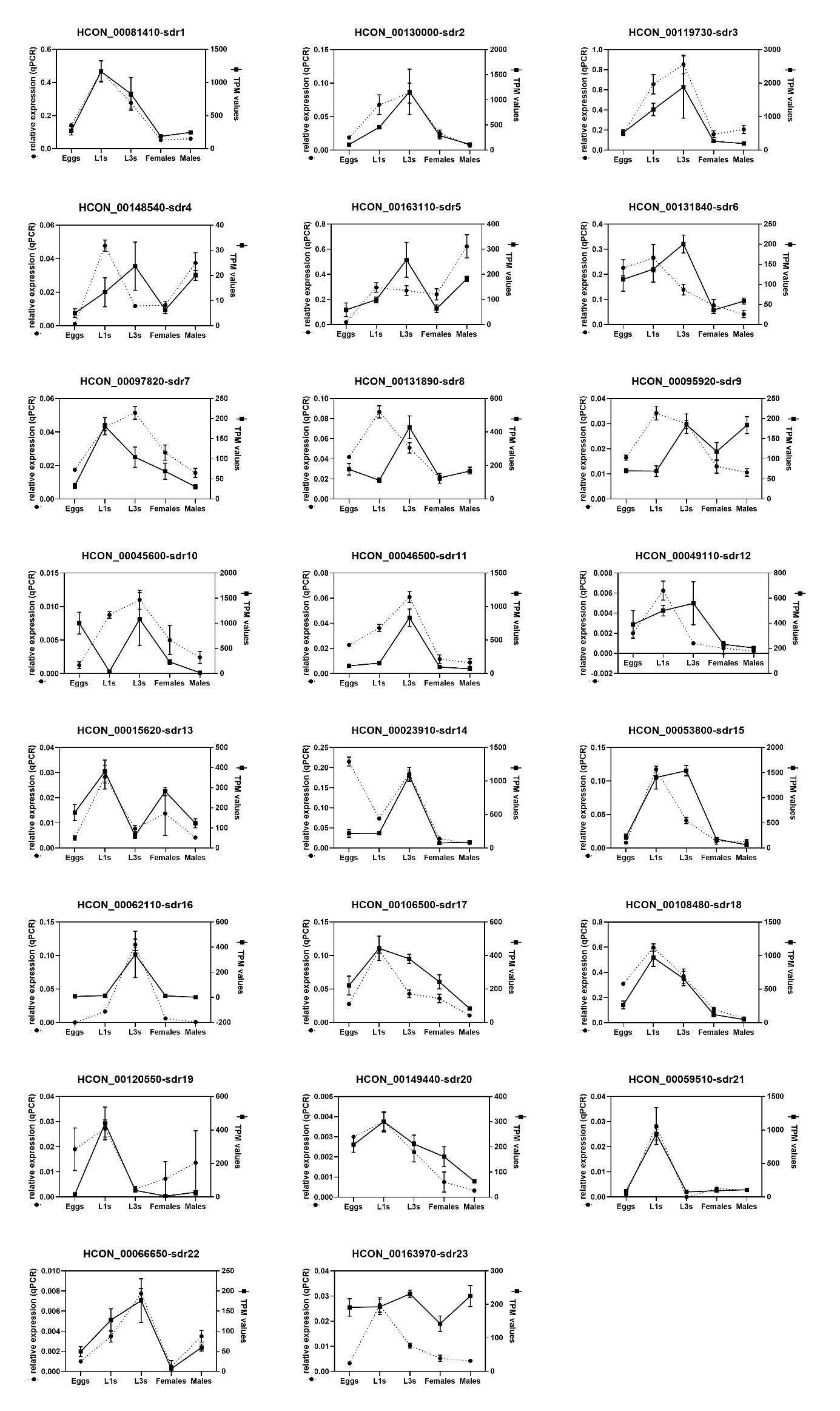
**
